# Supplementary material for: Activity in Group-Housed Home Cages of Mice as a Novel Preclinical Biomarker in Oncology Studies
Source: Cancers (Basel). 2023 Sep 29;15(19):4798. doi: 10.3390/cancers15194798 (PMC10571829; doi:10.3390/cancers15194798)
Supplement: Supplementary file 1 [file cancers-15-04798-s001.zip › cancers-2603255-supplementary.docx]

Supplementary Materials

**Table S1.** Summary table of the *p*-values obtained for the analysis of each treated group versus vehicle group at each day with the different biomarkers.

| **Group** | **Marker** | **Day** | | | | | | | | | | | | |
| --- | --- | --- | --- | --- | --- | --- | --- | --- | --- | --- | --- | --- | --- | --- |
|  |  | **1** | **2** | **3** | **4** | **5** | **6** | **7** | **8** | **9** | **10** | **11** | **12** | **13** |
| **Cisplatin 3 mg/kg** | **BW** | *0.0774* | *0.0774* | *0.0774* | **0.0006** | **0.0009** | *0.0619* | *0.0774* | **0.0012** | **<0.0001** | **0.0002** | **<0.0001** | **<0.0001** | **<0.0001** |
|  | **DVC® activity** **(dark phase)** | 0.3923 | 0.3923 | **0.0030** | 0.3923 | 0.3923 | 0.3923 | **0.0081** | **0.0032** | *0.0534* | **<0.0001** | **<0.0001** | **0.0030** | **0.0038** |
|  | **DVC®** **activity** **(light phase)** | 0.6712 | 1.0000 | 1.0000 | 1.0000 | 1.0000 | 1.0000 | 0.3224 | 1.0000 | 0.3224 | 1.0000 | 1.0000 | 0.5296 | 1.0000 |
|  | **DVC® activity** **(day)** | 0.1014 | 0.4600 | *0.0703* | 0.4600 | 0.4600 | 0.4600 | **0.0012** | **0.0034** | **0.0041** | **<0.0001** | **<0.0001** | **0.0009** | **0.0041** |
|  | **Clinical Score** | **0.0119** | 1.0000 | 1.0000 | 0.2177 | 0.2318 | 0.1361 | **<0.0001** | **<0.0001** | **<0.0001** | **0.0021** | **<0.0001** | **<0.0001** | **<0.0001** |
| **CPP  100 mg/kg** | **BW** | 0.3449 | **<0.0001** | **<0.0001** | **<0.0001** | **<0.0001** | **<0.0001** | **<0.0001** | **<0.0001** | **<0.0001** | **<0.0001** | **<0.0001** | **<0.0001** | **<0.0001** |
|  | **DVC®** **activity** **(dark phase)** | 0.9821 | 0.9821 | 0.1129 | 0.9821 | 0.9821 | 0.9821 | 0.1425 | 0.2725 | 0.7262 | **0.0131** | 0.4339 | 0.9821 | 0.6649 |
|  | **DVC®** **activity** **(light phase)** | 0.1307 | 0.9984 | 0.9984 | 0.9984 | 0.9984 | 0.9984 | 0.9984 | 0.9984 | 0.9984 | 0.9984 | 0.9984 | 0.9984 | 0.9984 |
|  | **DVC® activity** **(day)** | 0.4394 | 0.9960 | 0.5339 | 0.9960 | 0.9960 | 0.9960 | 0.0970 | 0.3283 | 0.8881 | 0.2337 | 0.5062 | 0.8881 | 0.4215 |
|  | **Clinical Score** | **0.0066** | 1.0000 | 1.0000 | 0.2722 | **0.0239** | **0.0239** | **<0.0001** | **<0.0001** | 0.3091 | 0.5555 | **<0.0001** | **<0.0001** | **<0.0001** |

*p*-values are shown for dark phase, light phase, and the whole day, respectively. *p*-values highlighted in red and bold type are significant *p*-values (*p* < 0.05) and *p*-values in orange and italics are *p*-values closed to significance; BW, body weight; CPP, cyclophosphamide.


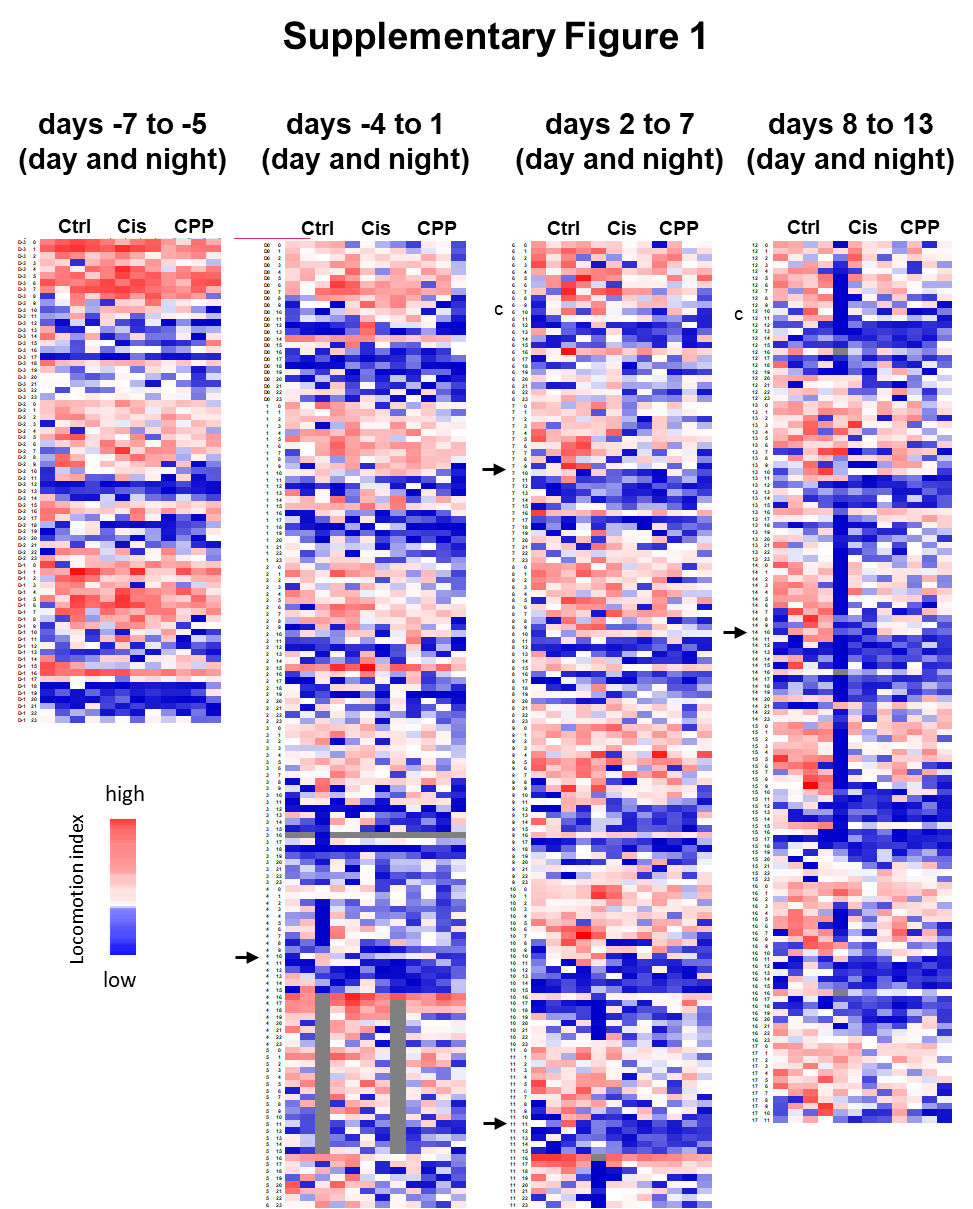


**Figure S1.** Heatmaps generated with locomotion index values displayed in 1-hour intervals (each row) depicting activity patterns of mice in the different study groups, with each column representing a DVC unit housing 4 mice. Colors denote the levels of calculated locomotion index, from blue (reduced locomotion index) to red (high locomotion index). The grey color indicates time slots for which no data were collected. Arrows denote treatment intervention, and “c” cage change.


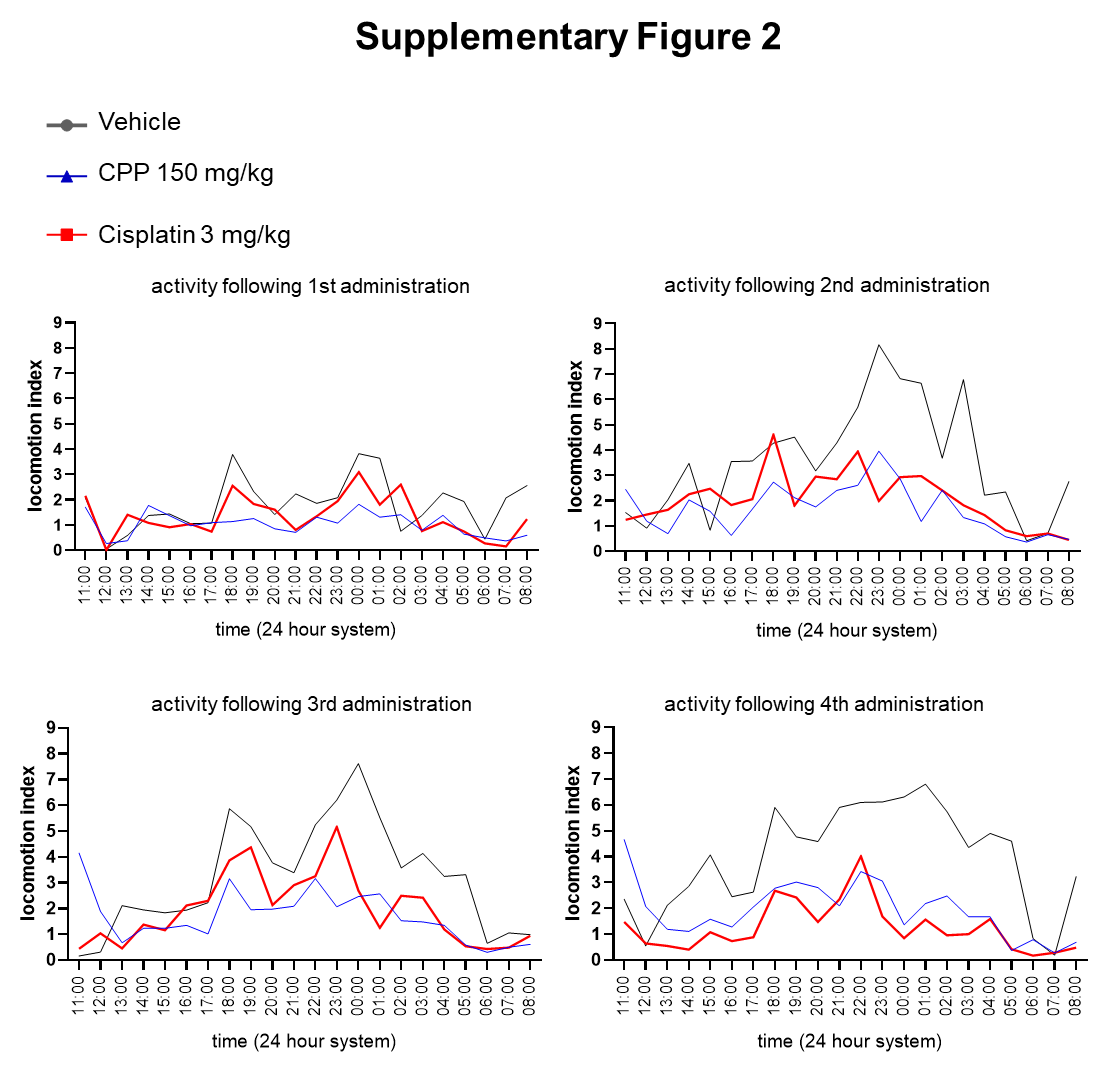


**Figure S2.** Curves depicting the locomotion index in the hours post administration of the compounds, or the control vehicle. Values are presented as mean values collected from the 4 cages of each group. For sake of readability, standard deviations are not shown.
